# The Cdk inhibitor dinaciclib as a promising anti-tumorigenic agent in biliary tract cancer

**DOI:** 10.1080/15384047.2024.2439057

**Published:** 2024-12-12

**Authors:** Celina Ablinger, Daniel Neureiter, Theresa Mähr, Christian Mayr, Tobias Kiesslich, Nicole Maeding, Irina Valenta, Maximilian Ardelt, Fabian Wilhelm, Elen Neureiter, Markus Ritter, Johanna Pachmayr, Petra Huber-Cantonati

**Affiliations:** a Institute of Pharmacy, Department of Pharmaceutical Biology and Clinical Pharmacy, Paracelsus Medical University, Salzburg, Austria; b Center for Physiology, Pathophysiology and Biophysics, Institute for Physiology and Pathophysiology Salzburg, Paracelsus Medical University, Salzburg, Austria; c Institute of Pathology, Paracelsus Medical University/University Hospital Salzburg (SALK), Salzburg, Austria; d Cancer Cluster, Salzburg, Salzburg, Austria; e Department of Internal Medicine I, Paracelsus Medical University/University Hospital Salzburg (SALK), Salzburg, Austria; f Cell Therapy Institute, Spinal Cord Injury and Tissue Regeneration Centre Salzburg (SCI-TReCS), Paracelsus Medical University, Salzburg, Austria; g Ludwig Boltzmann Institute for Arthritis and Rehabilitation, Paracelsus Medical University, Salzburg, Austria; h Gastein Research Institute, Paracelsus Medical University, Salzburg, Austria; i Kathmandu University School of Medical Sciences, Dhulikhel, Nepal

**Keywords:** biliary tract cancer, Cdk, dinaciclib, cytotoxicity, apoptosis, 3D cell culture

## Abstract

Biliary tract cancer (BTC) is a rare malignancy with rising incidence. The therapeutic options are limited and the overall survival remains poor. Cyclin-dependent kinases, drivers of cell cycle and transcription have numerous biological functions and are known to be dysregulated in numerous tumor entities. Dinaciclib is a selective Cdk1/2/5/9 inhibitor with anti-tumor activity. In the present study, the efficacy of dinaciclib was tested on a comprehensive BTC cell-line model. The results indicate a heterogeneous expression pattern of Cdk1/2/5/9, as well as various differentiation tumor markers in BTC cells. We demonstrated that dinaciclib reduces cell viability, ATP levels, and proliferation rates. Moreover, dinaciclib induces apoptosis via increased caspase 3/7 activity and reduced expression levels of the anti-apoptotic protein Mcl-1 in a concentration- and cell line -dependent manner. 3D cell culture confirms the cytotoxic impact of dinaciclib under more physiologic tumor conditions. Additionally, dinaciclib affects different cell growth regulators like EGFR and STAT3 on gene and protein level, thus decreasing tumor growth. In summary, our study indicates that dinaciclib acts as a promising anti-tumorigenic agent in 2D and 3D *in vitro* BTC models and thus encourages further investigation.

## Introduction

Biliary tract cancer (BTC) is a rare malignancy with dismal outcome due to a lack of efficient therapies and frequently occurring recurrence. BTCs are originally classified due to their anatomical/tissue localization, whereas the generic term BTC describes both intrahepatic and extrahepatic bile duct carcinoma as well as gallbladder carcinoma.^1,2^ Although the incidence of BTC is low in the Western world, global mortality for intrahepatic cholangiocarcinoma has remarkably increased in the last decades.^1,3,4^ So far, complete resection of the organ is the first option for intrahepatic cholangiocarcinoma. However, the median survival rate after surgical resection is low. For advanced stages, a combination of cisplatin and gemcitabine medication is the standard of care.^4^ Targeted therapies (against IDH1 mutation or FGFR2 fusion) have been shown efficient but require molecular testing and only a few patients are eligible.^5^ Despite these various treatment options, the overall survival rate for BTC remains poor, mainly as a result of late diagnosis and disease recurrence.^3^ Therefore, novel therapeutic targets are highly needed for the treatment of BTC.

Dysregulated cell proliferation, one of the hallmarks of cancer, is often driven by alterations and unrestrained activation of Cdks.^6^ These Cdk dysregulations are noticed in many types of tumors, and therefore there was a growing interest in developing inhibitors targeting these cell-cycle proteins to inhibit cancer progression.

So far, several pan-Cdk inhibitors entered into clinical trials. Dinaciclib, a multi-target Cdk1, Cdk2, Cdk5 and Cdk9 second-generation inhibitor, leads to the arrest of cell-cycle progression and induces apoptosis in various tumor cells.^4,7^ *In vivo* studies indicate that dinaciclib indeed has anti-tumor effects and reduces tumor growth.^4,8^ Saqub et al. already showed that dinaciclib is able to prevent proliferation of tumor cells *in vitro* and *in vivo*.^4^ Furthermore, clinical trials reveal that dinaciclib is well tolerable in patients with less side effects than other multi kinase inhibitors. Therefore, targeting or inhibition of Cdk activity provides a potential novel therapeutic approach for several types of tumors, including BTC, and could be used to improve conventional anti-cancer treatments if administered in combination.^9,10^

Previous data have demonstrated that dinaciclib effectively suppresses the growth of BTC cells and induces apoptosis *in vitro* and *in vivo*. Additionally, cell-cycle analysis showed that dinaciclib reduces cell proliferation via a decrease in the S-phase population and lowers the gene expression of anti-apoptotic proteins in BTC.^4^ However, despite the promising initial data, further investigation of dinaciclib is required to gain deeper insights into its mechanistic effects and to evaluate its potential as a treatment option for BTC.

In the present study, we hypothesize anti-proliferative and cytotoxic effects of dinaciclib in a 2D as well as 3D BTC cell culture model. Moreover, we assume that the growth suppression is connected to the inhibition of various tumor-promoting growth factor signaling pathways. Therefore, the aim of the present study was to elucidate the anti-tumorigenic effect of dinaciclib and the underlying mode of action in various BTC cell lines.

## Results

### Dinaciclib reduces the viability of BTC

**Figure 1.**
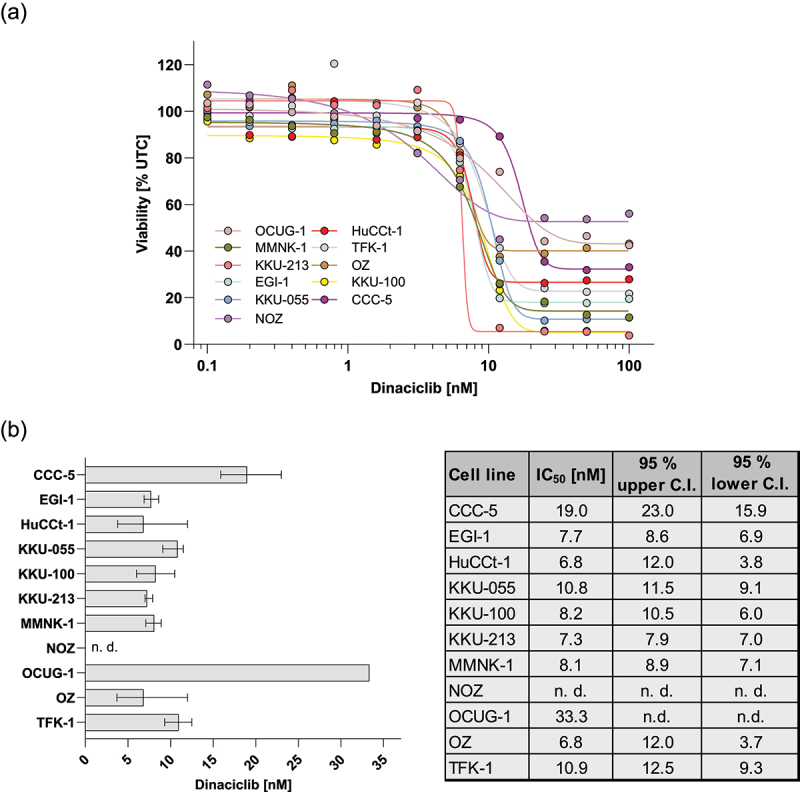
Dinaciclib decreases cell viability dose-dependent in biliary tract cancer (BTC) and one non-tumorigenic cell line. (a) Cell viability of ten malignant BTC cell lines and one non-tumorigenic (MMNK-1) cell line after treatment with increasing dinaciclib concentrations for 72 h. Lines represent the overall fit of the four-parameter logistic regression. (b) IC_50_ values including 95% confidence interval (C.I.) were calculated using four-parameter logistic regression. n. d. = not determined.

To get a better understanding of the effect of dinaciclib, we tested the cytotoxicity on ten malignant BTC and one non-tumor cholangiocyte (MMNK-1) cell line. Dinaciclib significantly reduced BTC cell viability in a dose-dependent manner at low nM concentrations (Figure 1a) with a specific sensitivity pattern between cell lines: sensitive (EGI-1, HuCCt-1, KKU-055, KKU-100, KKU-213, MMNK-1, OZ, TFK-1) and less dinaciclib-sensitive (CCC-5, OCUG-1; for NOZ cells, no IC_50_ could be determined, as the viability did not drop below 50%) cells with IC_50_ values ranging from 6.8 to 33.3 nM (Figure 1b). Furthermore, at concentrations ≥12.5 nM, a viable cell fraction ranging from 10% to up to 55% remained, even at the highest dinaciclib concentration of 100 nM. Therefore, we assume that these cells were resistant toward dinaciclib treatment resulting in a high remaining viable fraction. The corresponding significances for each treatment versus control are presented in Table S1.

### Cdk1/2/5/9 are heterogeneously expressed in BTC cell lines and Cdk5 positively correlates with IC_50_

**Figure 2.**
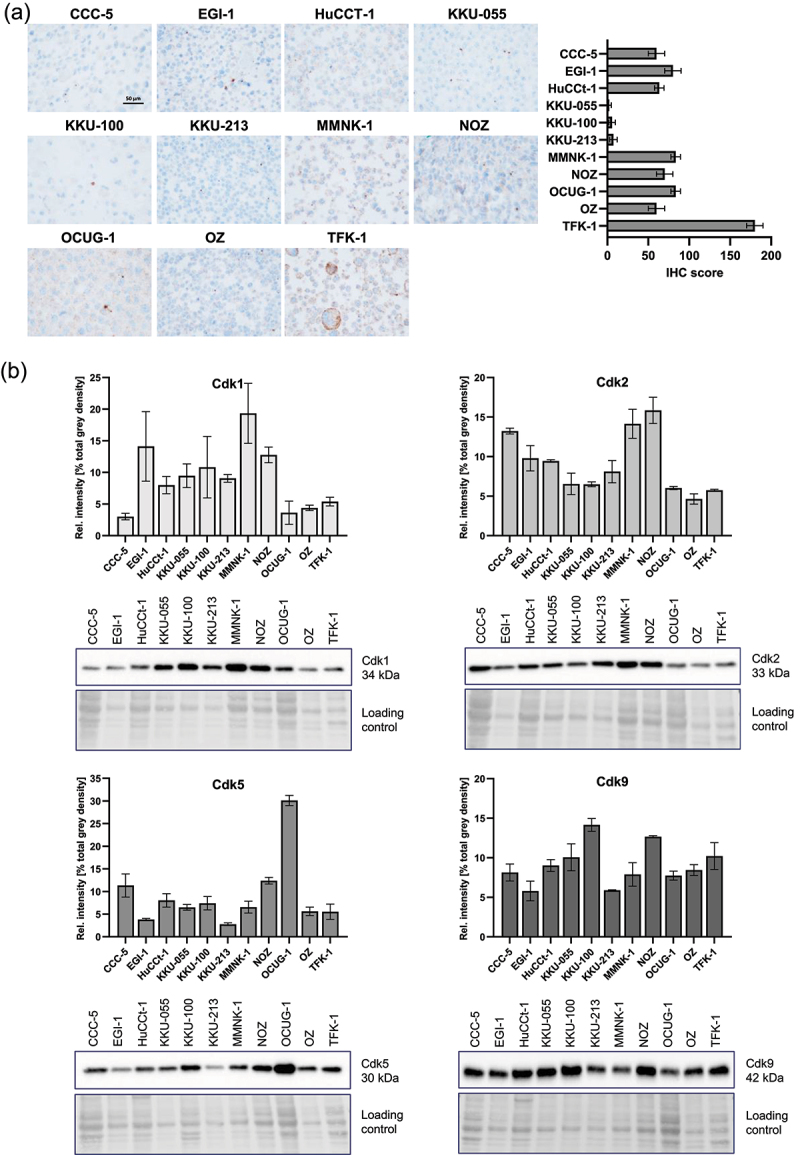
Cdks, especially Cdk5, are differently expressed in various biliary tract cancer (BTC) cell lines. (a) Representative immunohistochemical staining of Cdk5 in all eleven used (ten BTC cell lines and one non-tumorigenic biliary cell line) naïve (untreated) cell lines (magnification 400×) showing heterogeneous expression levels of Cdk5. The expression levels of Cdk5 range from very low (less than 10 for KKU-055, KKU-100, KKU-213), medium (between 50 and 100 for CCC-5, EGI-1, HUCCt-1, MMNK-1, NOZ, OCUG-1 and OZ) to high (more than 150 for TFK-1), indicating the heterogeneity of Cdk5 protein distribution. Data are presented as mean ± SEM of three different areas of the cell blocks. (b) Cdk1, Cdk2, Cdk5 and Cdk9 levels were analyzed with immunoblot in BTC and non-tumorigenic (MMNK-1) cells. Protein expression levels of cells were normalized to the sum of gray density of each biological replicate. Representative immunoblots of BTC cells probed with antibodies against Cdk1, 2, 5 and 9 are shown. Expression is quantified as mean value ± SEM based on *n* = 3 biological replicates.

To evaluate the basal Cdk5 expression, IHC stainings (Figure 2a) were performed on the BTC cell-line panel. IHC stainings revealed high Cdk5 expression in TFK-1 cells, and low expression was observed in KKU-055, KKU-100 and KKU-213 cells. Remaining cell lines possessed a moderate Cdk5 staining with mean IHC scores from 60 to 83. Furthermore, immunoblot analyses revealed the heterogenous expression of Cdk1, 2, 5 and 9 in BTC and MMNK-1 cell lines: High Cdk5 expression was present in OCUG-1 cells, and the highest expression of Cdk9 was observed in KKU-100 cells. Cdk1 and Cdk2 showed the highest expression in EGI-1, MMNK-1 and NOZ and CCC-5, MMNK-1 and NOZ, respectively (Figure 2b).

**Figure 3.**
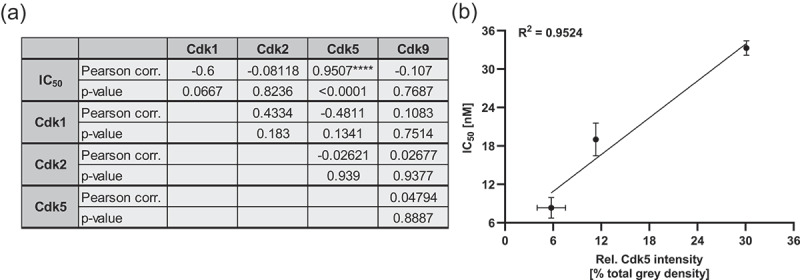
Cdk5 expression correlates with the IC_50_ values in biliary tract cancer (BTC). (a) Pearson correlation analysis between the expression of Cdks and IC_50_ values or between the Cdks itself, ****p<.1, n = 3. (b) Corresponding dot plot with a linear fit for the correlation of the Cdk5 expression (displayed as rel. intensity [% total gray density]) and respective IC_50_ values. For better illustration, IC_50_ and Cdk5 intensities of EGI-1, HuCCt-1, KKU-055, KKU-100, KKU-213, MMNK-1, OZ and TFK-1 were grouped and depicted as one dot including SEM.

While the results of IHC and immunoblotting were not identical, immunoblot correlation analysis of all cell lines revealed a highly significant positive correlation between Cdk5 protein expression and IC_50_ values of dinaciclib (Figure 3a correlation plot Figure 3b).

### Dinaciclib reduces cell proliferation and induces apoptosis in BTC cells

For subsequent experiments, the cell lines KKU-100, OCUG-1 and OZ were used. Selection was based on the specific sensitivity pattern to the dinaciclib treatment: KKU-100 exhibited a high sensitivity toward dinaciclib, as represented by a low IC_50_ value (8 nM, Figure 1b) and a low cell viability (Figure 1a). In contrast, OCUG-1 cells were less sensitive, which is indicated by a high IC_50_ value (33 nM, Figure 1b) and a large fraction of viable and thus resistant cells (Figure 1a). OZ cells displayed a high sensitivity toward dinaciclib with a low IC_50_ value (7 nM Figure 1b) and a large fraction of resistant cells (Figure 1a).

For a general comparison of dinaciclib treatment in DMEM with 10% serum or serum-free DMEM (sfDMEM), we additionally tested the effect of dinaciclib on BTC cells in serum containing media. We observed that the trend in cytotoxic effects was similar to the experiments with sfDMEM (Figure S1). Therefore, all subsequent experiments were conducted using sfDMEM.

**Figure 4.**
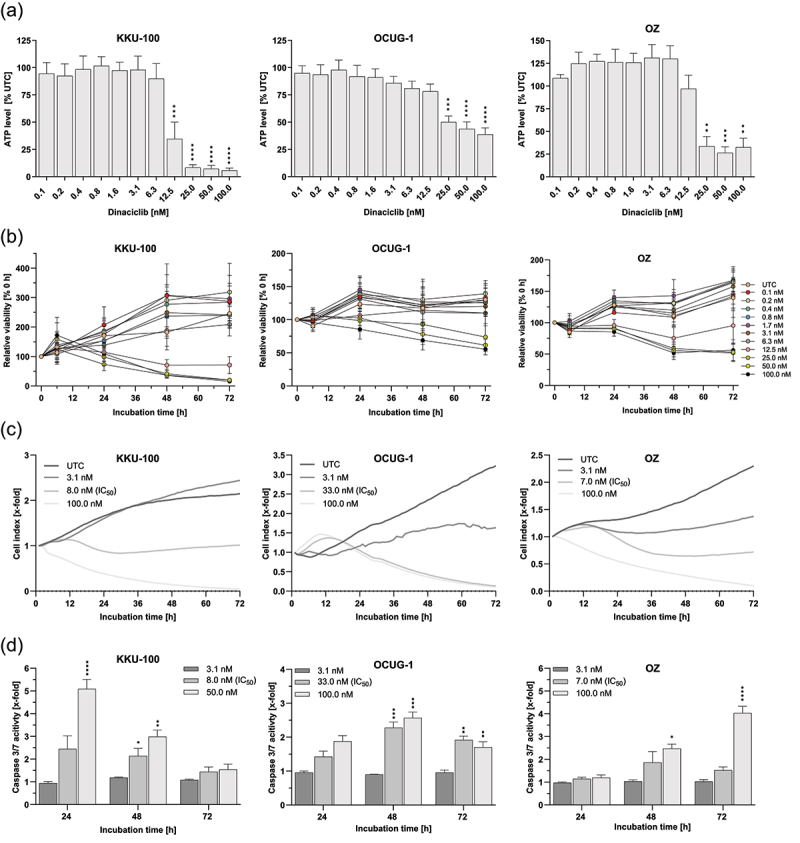
Dinaciclib decreases viability, ATP levels, proliferation and induces caspase activity in biliary tract cancer (BTC). (a) ATP levels of KKU-100, OCUG-1 and OZ cells after treatment with dinaciclib concentrations ranging from 0.1–100 nM for 72 h are shown. ATP levels were normalized to the untreated control [% UTC = untreated control]. (b) Time-resolved analysis of KKU-100, OCUG-1 and OZ cell viability after treatment with dinaciclib. Viability was determined after 0, 6, 24, 48 and 72 h of incubation, respectively and normalized to the respective 0 h fluorescence values [% 0 h]. (c) Proliferation of KKU-100, OCUG-1 and OZ cells after treatment with 3.125 nM, corresponding IC_50_ or 100 nM dinaciclib. The cellular impedance signal was recorded continuously for 72 h using the unitless parameter cell index that is proportional to the cell number. Shown are representative curves of one biological replicate. (d) Caspase 3/7 levels were measured to detect apoptosis in KKU-100, OCUG-1 and OZ cells after treatment with 3.125 nM, IC_50_ and 50 nM dinaciclib for 24, 48 and 72 h. Luminescence values were normalized to UTC [x-fold]. Data are presented as mean values ± SEM, *n* = 3 and significances were tested using a one-way analysis of variance (ANOVA), Dunnett ****p < .05, *p < .01, **p < .001 and ***p < .0001.

To gain a better insight into the impact of dinaciclib on metabolic activity, ATP levels were measured 72 h after dinaciclib treatment. As shown in Figure 4a, dinaciclib significantly decreased the ATP levels in BTC cells. Again, KKU-100 cells exhibited low levels of ATP after treatment with 12.5 nM or higher dinaciclib concentrations. A significant decline in ATP levels was observed in OCUG-1 and OZ cells but only at higher concentrations (≥25 nM). Additionally, these cell lines again showed a high metabolic activity (Figure 4a) following dinaciclib treatment with 100 nM. Therefore, ATP data coincided with the dose-dependent cytotoxic effect shown in Figure 1a.

To determine whether the reduction in cell viability was anti-proliferative or direct cytotoxic, we performed a time-resolved analysis of the cell viability (Figure 4b). In KKU-100 cells, a clear cytotoxic effect was observed for concentrations higher than 12.5 nM after 24 h and cell viability further declined after 48 and 72 h. A clear trend was determined for OCUG-1 cells, concentrations over 25 nM led to a weak reduction of cell viability after 24 h, but viability was stronger reduced after 48 and 72 h incubation with dinaciclib. Similarly, dinaciclib at concentrations higher than 25 nM result in a cytotoxic effect after 48 and 72 h. Again, a resistant cell fraction of OCUG-1 and OZ cells was still present after 100 nM dinaciclib treatment for 72 h. These results indicate that the effect of dinaciclib was largely anti-proliferative at low nM concentrations but the cytotoxicity increased at higher concentrations.

To confirm the anti-proliferative effect of dinaciclib, the proliferation ability of BTC cells following dinaciclib treatment was assessed. KKU-100, OCUG-1 and OZ cells were treated with 3.125 nM (highest non-lethal concentration), the respective IC_50_ (8 nM for KKU-100, 33 nM for OCUG-1 and 7 nM for OZ), 100 nM (highest lethal concentration) or left without any treatment and the cellular impedance (corresponding to cell proliferation) was continuously recorded for 72 h. The results revealed that cell proliferation was significantly reduced after dinaciclib treatment in BTC cells (Figure 4c). As expected, low-dose dinaciclib (3.1 nM) was not anti-proliferative. Treating the cells with dinaciclib concentrations corresponding to their respective IC_50_ led to a proliferation stop in KKU-100 cells and a reduction in cell proliferation in OZ cells. In OCUG-1 cells, the IC_50_ dinaciclib concentration as well as the 100 nM dinaciclib treatment in KKU-100, OCUG-1 and OZ cells led to a clear drop of the cell index below initial values thus indicating a direct cytotoxic effect. Caspase 3/7 measurement demonstrated apoptosis induction in BTC cells in a dose-dependent manner. Treatment of cells with the highest non-lethal concentration (3.1 nM) did not lead to an increase in caspase 3/7 activity, whereas higher concentrations led to a marked rise following dinaciclib treatment.

Additionally, microscopy analyses showed that dinaciclib treatment led to morphological changes in cell shape of the BTC cell lines KKU-100, OCUG-1 and OZ (Figure S3). The highest concentration (50 nM KKU-100, 100 nM OCUG-1 and OZ) especially caused a tremendous change in the cell morphology, whereby shrinkage of the cells is a morphologically characteristic of apoptosis.^11^

### Dinaciclib declines the size and viability of 3D BTC spheroids

**Figure 5.**
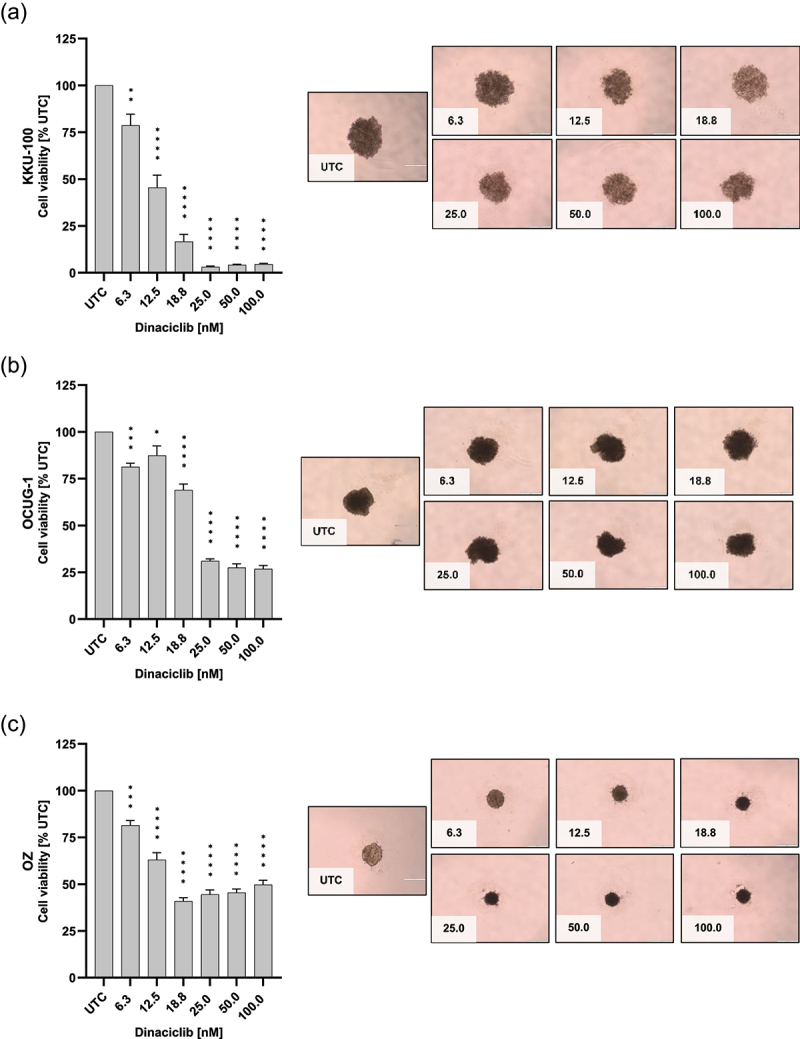
Dinaciclib affects the proliferation and size of 3D biliary tract cancer (BTC) spheroids. Viable (A) KKU-100, (B) OCUG-1 and (C) OZ spheroid cells after 96 h stimulation with the indicated dinaciclib concentrations measured with the Promega CellTiter-Glo® 3D cell viability assay. Exemplary pictures of each treatment condition of the different cells are presented (scale bar 500 µm). Values shown indicate concentrations in nM. Data are presented as mean values ± SEM, n = 4 and significances were tested using a one-way analysis of variance (ANOVA), Dunnett’s compared to the untreated control, ****p<.05, *p <.01, **p <.001, ***p <.0001.

Next, we assessed the effect of dinaciclib in 3D BTC spheroids of all ten BTC and one non-tumorigenic cell line (Figure 5 and Figure S4 and S5). We observed a significant reduction in cell viability in most BTC cell lines spheroids following a 96 h dinaciclib treatment except HuCCt-1. Notably, the effects of dinaciclib were concentration-dependent in most cases. Dinaciclib also had varying effects on the shape and size of the spheroids. In some cell lines (Figure S4 and S5: CCC-5, KKU-213 and MMNK-1), cells started to detach from the spheroids, leading to their dissolution after 96 h of treatment. In other cell lines (Figure S4 and S5: KKU-055, NOZ, TFK-1; Figure 5: OZ), a shrinkage of the spheroid occurred, while some lost their distinct boundaries and exhibit deformation.

In the representative cell-line KKU-100, the viability of spheroidal cells dropped to 5% compared to control following treatment with dinaciclib concentrations higher than 25 nM. We observed a cytotoxic effect of dinaciclib in OCUG-1 spheroids at concentrations higher than 25 nM, which was indicated by a cell viability of about 30% compared to control. In OZ spheroids, cell viability remained at about 50% after treatment with dinaciclib concentrations over 18.75 nM. It could thus be shown that KKU-100 spheroids were most sensitive toward dinaciclib, while OCUG-1 and OZ spheroids were less sensitive. However, the cell viability could not be measured for KKU-055 spheroids due to the size of the control spheroids (Figure S 5D).

### Dinaciclib affects the expression of proliferation and tumor progression-related genes

Uncontrolled cell proliferation is often driven by excessive and aberrant activation of growth receptors. Among these receptors, the EGFR pathway and its downstream targets ERK, protein kinase B (AKT) and STAT3 play a crucial role in signaling cascades that lead to the abnormal activation of genes, promoting uncontrolled cell growth and the progression of tumors.^12^

As dinaciclib reduced the proliferation ability and cell viability of BTCs in a 2D as well as in a 3D cell model, we aimed to elucidate how dinaciclib affects the gene expression of different proliferation- and tumor progression-related genes.

**Figure 6.**
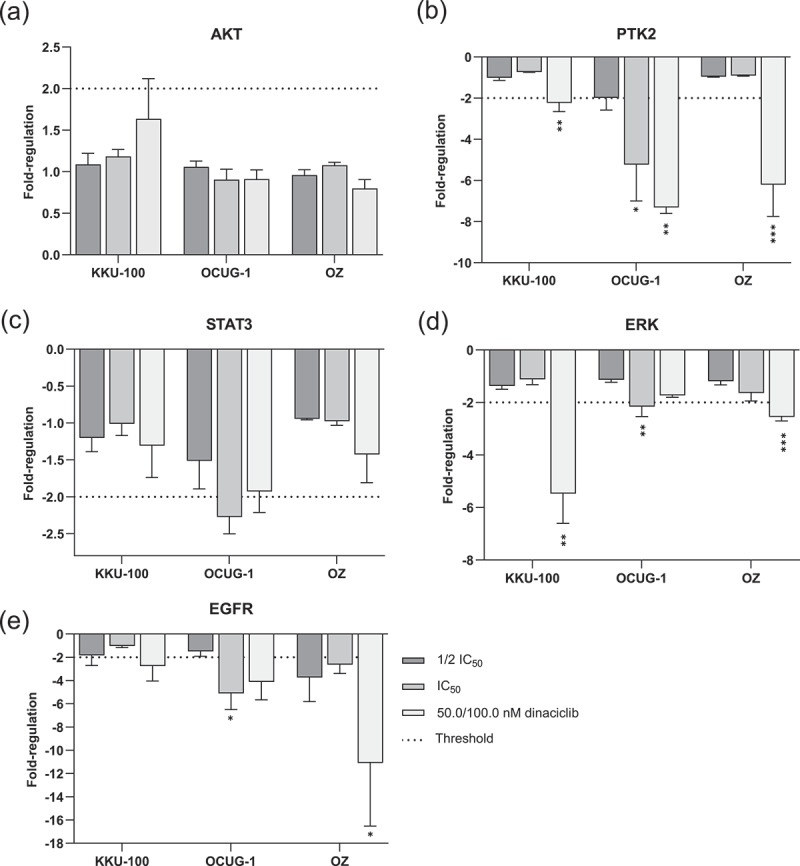
Dinaciclib affects the expression of genes involved in cell proliferation and tumor progression. The expression of (a) AKT, (b) PTK2, (c) STAT3, (d) ERK and (e) EGFR from treated biliary tract cancer (BTC) cells KKU-100, OCUG-1 and OZ cells is shown each relative to untreated samples. mRNA levels were analyzed following dinaciclib treatment with the respective ½ IC_50_ (KKU-100: 4 nM, OCUG-1: 16.5 nM, OZ: 3.5 nM), IC_50_ (KKU-100: 8 nM, OCUG-1: 33 nM, OZ: 7 nM) and 50 nM (KKU-100) or 100 nM (OCUG-1 and OZ) for 24 h. Transcript levels were quantified using the delta delta CT method, whereas mRNA levels were first referred to the housekeeping gene GAPDH and treated samples were further normalized to controls. The fold-regulation is the negative inverse of the fold-change, and the respective thresholds for up- and downregulation are depicted as dotted lines. Data are presented as mean values ± SEM, *n* = 3 and significances were tested for each dinaciclib concentration against controls using a one-way analysis of variance (ANOVA), Dunnett ****p < .05, *p < .01, **p < .001 and ***p < .0001.

As demonstrated in Figure 6, AKT and STAT3 transcripts of KKU-100, OCUG-1 and OZ cells were not significantly influenced by dinaciclib. Conversely, the expression of the focal adhesion kinase gene (PTK2), a downstream target of Cdk5, was consistently reduced in the BTC cell lines following treatment with dinaciclib (Figure 6b). As shown in (Figure 6d), dinaciclib significantly reduced the mRNA levels of ERK with the highest effect on the sensitive cell-line KKU-100. Furthermore, dinaciclib suppressed transcripts of EGFR using concentrations corresponding to the respective IC_50_ values or the highest dinaciclib concentration (50 nM KKU-100, 100 nM OCUG-1 and OZ, Figure 6e). As expected, dinaciclib treatment at low concentrations (½ IC_50_) had no effect on the gene expression (Figure 6a-e).

### Dinaciclib reduces EGFR and STAT3 expression in BTC cells

To confirm the effects of dinaciclib on gene expression, the protein levels of AKT, PTK2, STAT3, ERK and EGFR were determined.

**Figure 7.**
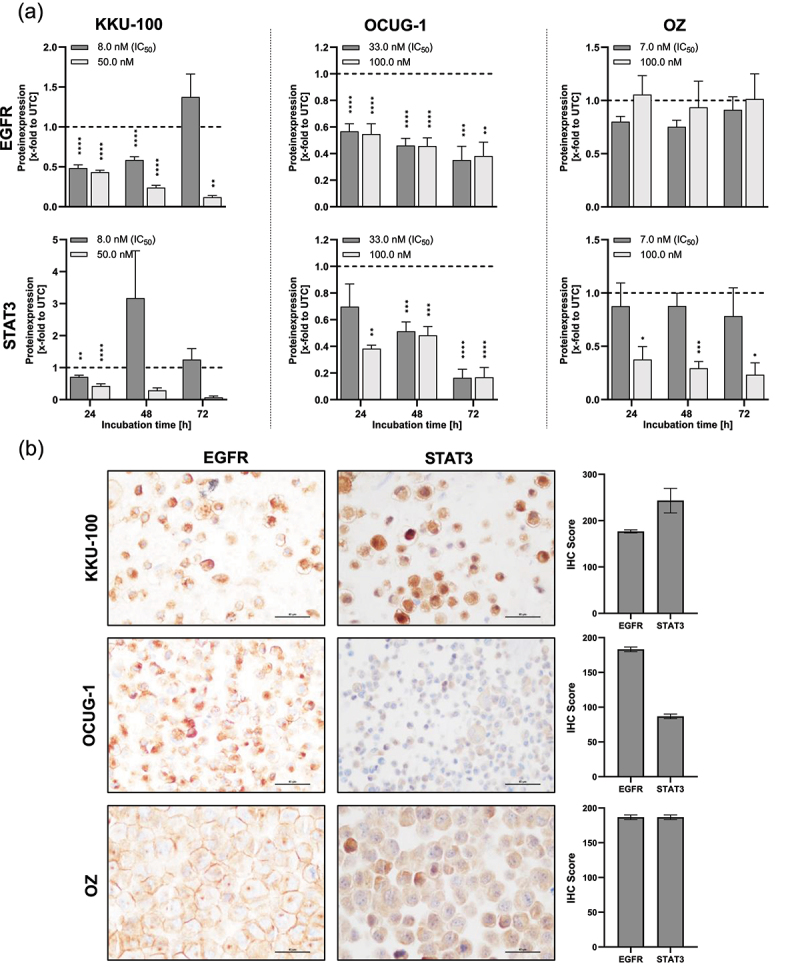
Dinaciclib affects the expression of proteins involved in cell proliferation and tumor progression. (A) Effect of dinaciclib on EGFR and STAT3 protein expression in KKU-100, OCUG-1 and OZ cells. Biliary tract cancer (BTC) cells were either treated with the corresponding IC_50_ (KKU-100: 8 nM, OCUG-1: 33 nM, OZ: 7 nM), 50 nM (KKU-100) or 100 nM (OCUG-1 and OZ) dinaciclib for 24, 48 and 72 h, respectively. Protein expression was quantified via normalization to corresponding loading controls (figure S7), which were further referred to untreated control cells [x-fold to untreated control = UTC]. Data are presented as mean values ± SEM, *n* = at least 3 and significances were tested for each dinaciclib concentration against controls using a one-way analysis of variance (ANOVA), Dunnett ****p < .05, *p < .01, **p < .001 and ***p < .0001. (B) Representative immunohistochemical staining of EGFR and STAT3 in the three naïve (untreated) BTC cell lines KKU-100, OCUG-1 and OZ (magnification 400×) showing heterogeneous but overall medium to high expression levels of EGFR and STAT3 in the three BTC cell lines. Data are presented as mean ± SEM of three different areas of the cell blocks.

For AKT, ERK and PTK2, no differences in protein expression were observed (shown in Figure S6). However, protein levels of EGFR and STAT3 were significantly decreased following dinaciclib treatment (Figure 7a). In the most sensitive cell-line KKU-100, we observed the highest effect of dinaciclib in the STAT3 and EGFR expression, whereby the protein levels were significantly reduced after treatment for 24 h.

In the less sensitive cell-line OCUG-1, treatment of cells with 33 nM (IC_50_) and 100 nM for 24, 48 and 72 h resulted in a significant reduction of EGFR and STAT3 protein expression compared to controls. As EGFR and STAT3 function in signaling pathways, we also investigated their phosphorylated forms (Figure S8). We observed a significant increase in phosphorylated EGFR in KKU-100 and OCUG-1 cells. In contrast, phospho-EGFR levels were decreased in OZ cells following dinaciclib treatment. Additionally, we demonstrated that dinaciclib significantly reduced activated STAT3, as its phosphorylation status was decreased in all of the tested cell lines.

Interestingly, for OZ cells, while STAT3 was downregulated following dinaciclib treatment, the substance had no effect on the EGFR levels. Representative immunoblots are presented in Figure S7. The discrepancies in mRNA and protein expression of STAT3 are potentially due to posttranslational regulations or differences in the turnover rate of mRNA and protein.^13^

Based on the promising effects of dinaciclib on EGFR and STAT3 expression, these proteins were additionally analyzed via IHC. However, no obvious correlations between IHC and immunoblot analyses are observable. Representative IHC staining and corresponding IHC scores are shown in (Figure 7b). EGFR and STAT3 were heterogeneously expressed in the three BTC cell lines studied, KKU-100, OCUG-1 and OZ, with a mean expression level of 177, 183 and 187 for EGFR and 243, 87 and 187 for STAT3, with the cell-line OZ showed quite similar expression levels of EGFR and STAT3 compared to KKU-100 (EGFR < STAT3) and OCUG-1 (EGFR > STAT3).

### Dinaciclib decreases the levels of Mcl-1 and reduces its phosphorylation at Ser64

**Figure 8.**
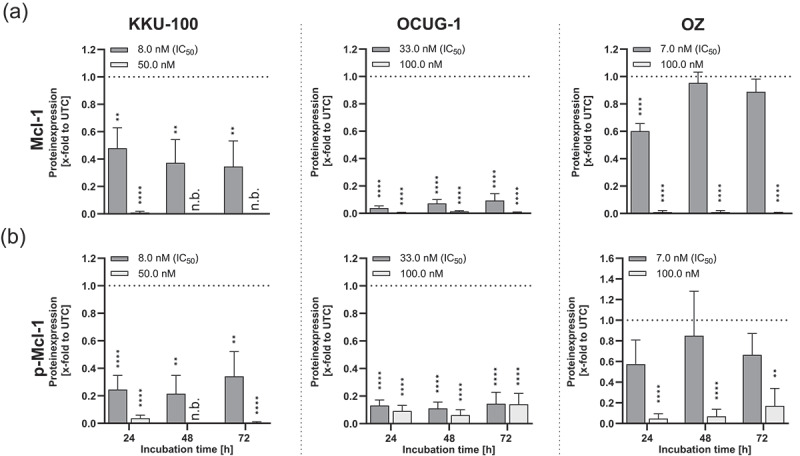
Dinaciclib reduces the expression of the anti-apoptotic protein Mcl-1 and the phosphorylation at Ser64. Effect of dinaciclib on (A) Mcl-1 and (B) phospho-Mcl-1 (Ser64) protein expression in KKU-100, OCUG-1 and OZ cells. Biliary tract cancer (BTC) cells were either treated with the corresponding IC_50_ (KKU-100: 8 nM, OCUG-1: 33 nM, OZ: 7 nM), 50 nM (KKU-100) or 100 nM (OCUG-1 and OZ) dinaciclib for 24, 48 and 72 h, respectively. Protein expression was quantified via normalization to corresponding loading controls (figure S7), which were further referred to untreated control cells [x-fold to untreated control = UTC]. Data are presented as mean values ± SEM, *n* = at least 3 and significances were tested for each dinaciclib concentration against controls using a one-way analysis of variance (ANOVA), Dunnett ****p < .05, *p < .01, **p < .001 and ***p < .0001.

Since dinaciclib is known to inhibit Cdk9, which affects the RNA Polymerase II and subsequently the transcription of anti-apoptotic genes, we examined the protein levels of the anti-apoptotic protein member Mcl-1 and its phosphorylation at S64 (Figure 8). In all the tested cell lines, treatment with 100 nM dinaciclib resulted in almost no detectable Mcl-1 signals. Treating the cells with their respective IC_50_ led to a significant reduction of Mcl-1 levels in KKU-100 and OCUG-1 cells (Figure 8a). We also investigated the phosphorylation status of Mcl-1 following treatment and found that phosphorylation at Ser64 was either significantly reduced or completely inhibited after treatment (Figure 8b). Flow cytometry analysis of Annexin V/7-AAD further confirmed apoptotic effects, showing an increase in the early apoptotic cell population in dinaciclib treated cells compared to controls, as shown in Figure S9.

## Discussion

In the present study, we tested the Cdk1/2/5/9 inhibitor dinaciclib on a comprehensive BTC cell-line model of ten BTC and one non-tumorigenic cell lines. We found that treatment of dinaciclib reduces the cell viability of BTC cells in a dose-dependent manner with IC_50_ values in a low nM range. Notably, BTC cells with the highest IC_50_ originated from the gallbladder, probably implicating that these tumors require a higher dose of dinaciclib than BTC cells of other origins. Likewise, Saqub et al. demonstrated a significant decline in cell growth in the cholangiocarcinoma cell-line HuCCt-1 following treatment at low nM concentrations.^4^ Our data is also in line with other studies, where it is reported that dinaciclib reduces cell growth at low nM dose in diverse tumor entities.^8,14,15^ In our study, the effect of dinaciclib was heterogeneous across BTC cells, therefore cell lines were grouped into sensitive and less sensitive cell lines, referring to their IC_50_. Notably, the non-cancerous cell-line MMNK-1 exhibited a response to dinaciclib. Since MMNK-1 is the only non-tumor cholangiocyte cell line commercially available, the heterogeneity is not given and thus not comparable with the comprehensive BTC cell-line model used in the study. Additionally, drawing a direct comparison remains challenging, as this specific cholangiocyte cell line was genetically modified to achieve immortality. Furthermore, as depicted in Figure S10, MMNK-1 cells also displayed a high sensitivity toward the standard chemotherapeutic cisplatin.

Interestingly, some cell lines retained a high viability even after highest dinaciclib treatment, which was referred to as resistant cells. Measuring of ATP levels following treatment confirmed the differences in response between cell lines and their metabolically active state of those cells. Such high levels of resistant and viable cells might be problematic for the development of resistances *in vivo*. However, to circumvent these potential resistance mechanisms, we would highly recommend to investigate combination therapies in BTC involving dinaciclib and, for example, cisplatin.

Additionally, we observed anti-proliferative and cytotoxic characteristics, depending on the substance dose, as concentrations in the low nM range led to a stop in proliferation and higher doses had cytotoxic impacts. Our data is in concordance with Howard et al. who also showed the anti-proliferative effect at lower concentrations (10 nM), but the cytotoxicity and induction of apoptosis at higher (40–80 nM) dinaciclib concentrations in endometrial cancer cells.^14^ Saqub et al. postulated an increased apoptotic population and caspase 3/7 activity following dinaciclib treatment in the CCA cell-line HuCCt-1 and one patient-derived xenograft (PDX) cell line.^4^ Thereby, dinaciclib is able to block cell-cycle progression via an accumulation of cells in the G2/M phase and a decrease in the G1 phase fraction and S-phase population, as reported by a study from Jane et al.^16,17^ Inhibition of Cdk1, a key player for progression into the M-phase, leads to a stop in proliferation and this cell-cycle arrest subsequently leads to cell death.^17,18^ This is also in line with the study of Saqub et al. that confirmed the decrease in S-phase population of CCA and PDX cells following dinaciclib treatment.^4^ While the inhibition of Cdk1 and Cdk2 led to a stop in proliferation, the inhibition of Cdk9 and thus of transcription, may result in apoptosis. Inhibition of Cdk9, the phosphorylation unit of polymerase II at Ser2, leads to a decreased transcription of anti-apoptotic proteins like Mcl-1 and Bcl-2, which subsequently induces apoptosis in endometrial cancer cells.^14^ This could also be shown in CCA cells, where dinaciclib treatment leads to a decrease in Bcl-xL and Bcl-2 expression and thus induces cell death.^4^ Our results support the findings of other researchers, as we demonstrated a decreased level of the anti-apoptotic protein Mcl-1 and reduced phosphorylation at Ser64 following dinaciclib treatment.^14^

As 3D spheroids better mimic the actual tumor property than 2D cultures, dinaciclib was tested on a 3D BTC cell-line model. Again, we were able to show the specific sensitivity pattern toward dinaciclib and cells that display a high viability following treatment. Notably, 3D cultures did not require higher concentrations of dinaciclib to achieve the same effect as monolayer although it is stated, that 3D cultures are less sensitive toward therapeutic substances.^19^

Protein levels of Cdk1/2/5/9, as molecular targets of dinaciclib, were heterogeneously expressed in the BTC cell- line panel. IHC staining of Cdk5 confirmed this heterogeneity, however the IHC data obtained did not exhibit a clear overlap with the baseline expression data derived from immunoblotting. Interestingly, the cell line OCUG-1 displayed the highest Cdk5 levels in the immunoblot method, whereas protein levels were only moderate in IHC staining and vice versa for TFK-1 cells. This might be explained by the two distinct methods and the variations in sample preparation. The discrepancies might also be due to different possible localization states of Cdk5 in the nucleus or cytoplasm. As described by Ino et al., it might occur that IHC antibodies do not recognize proteins on slides although they show positive signals by immunoblot because of the state and environment around the protein of interest. Additionally, Cdk5 may form complexes with other proteins and thus are not recognized by certain antibodies.^20^ It is also possible that post-translational modifications, which have not yet been demonstrated and associations with activators may explain the varying detection. Notably, we did not test whether dinaciclib affects its target genes, as Saqub et al. have already demonstrated the suppressed expression of Cdk2, Cdk5 and Cdk9 in CCA cell lines following dinaciclib treatment.^4^

Furthermore, we investigated additional differentiation markers related to the epithelial-to-mesenchymal transition (EMT) in order to draw conclusions about phenotypic characteristics. Our findings revealed that EMT markers such as E-cadherin and vimentin, as well as the general tumor marker Ki-67, are heterogeneously expressed in BTC cells (Figure S2). Nevertheless, no discernible correlation between dinaciclib and the mentioned differentiation markers was determinable (data not shown). However, we provided first insights into the EMT expression patterns of various BTC cell lines, concluding that most BTC cells express either the epithelial (E-cadherin) or the mesenchymal (vimentin) marker, but not both. Since EMT is involved in tumor metastasis and aggressiveness, it would be interesting to analyze patient samples with regard to the EMT expression pattern and further correlate these markers with tumor stage in the future.

Correlation analysis revealed a highly significant positive correlation between Cdk5 expression levels and dinaciclib IC_50_ values. This suggests that cells exhibiting high levels of Cdk5 require higher dosage of dinaciclib to elicit an equivalent response. Recognizing Cdk5 levels as a potential biomarker could have clinical implications, guiding personalized dosage strategies for better treatment outcomes. So far, no further data regarding the correlation between Cdk5 and dinaciclib sensitivity is available; however some studies have revealed that an increased expression of Cdk5 is more likely to be associated with chemo-resistance, whereas a lower expression is more likely to be associated with increased sensitivity to chemotherapy in ovarian cancer.^17^

Various mRNA levels of proliferation-related genes were analyzed following treatment, and we observed a significant reduction in PTK2, ERK and EGFR transcripts of BTC cells. This leads to the hypothesis that Cdk5 and/or Cdk9 might modulate transcription factors that are involved in the transcription of the mentioned genes. PTK2 is involved in growth factor signaling, and cell proliferation and activation of the PTK2 gene might be an important early step in cell growth and intracellular signal transduction pathways.^21^ Therefore, the inhibition of proliferation via dinaciclib might lead to a reduced transcription of PTK2. Unfortunately, no additional data is available regarding the effect of dinaciclib on transcriptional regulation. Protein levels of EGFR and STAT3 were significantly reduced following dinaciclib treatment, while other proteins remained unaffected (Figure S6). Saqub et al. indicated a suppression of Cdk2/5/9 levels after dinaciclib treatment.^4^ Therefore, we postulated that Cdk5 inhibition reduces the total amount of Cdk5 and this reduction leads to less EGFR expression and the associated signaling pathway. As EGFR functions in the signaling pathway, we analyzed its phosphorylated form, which revealed an increased phospho-EGFR status after dinaciclib treatment in two of the three cell lines tested. The accumulation of phosphorylated EGFR may result from Cdk inhibition, leading to a disrupted cell proliferation and potentially activating alternative survival pathways. In response to the cell-cycle inhibition, tumor cells may upregulate EGFR to bypass the growth inhibition caused by stress. To address this possible issue, combining dinaciclib with an EGFR inhibitor might serve as a more effective strategy to inhibit tumor growth.

It is also known that Cdk5 modulates STAT3 activation which subsequently leads to cell proliferation in thyroid cancer.^22^ Hsu et al. additionally showed that Cdk5 interacts with STAT3. This interaction depends on Cdk5 activation, whereby the activated STAT3 correlates with cell proliferation control and inhibition of Cdk5 leads to less interaction between STAT3 and Cdk5. In contrast to our study, Cdk5 inhibition with roscovitine reduced the levels of phosphorylated STAT3, while the amount of total STAT3 was unaffected. Although we observed lower total STAT3 levels, our data supports the finding of decreased activated STAT3, as we observed a reduction in STAT3 phosphorylation.^23^ Sang et al. revealed that EGFR-dependent Cdk5 triggers STAT3 signaling.^24^ Hence, if there is a reduced amount of EGFR, there is also a decrease in active Cdk5, leading to a reduction in active STAT3 signaling. Based on the promising effects of dinaciclib on EGFR and STAT3 levels, we examined these proteins in IHC. However, we could not establish any evident correlation between IHC data and immunoblot. While obvious differences in the intracellular localizations are present between the cell lines, no connection between the staining pattern and the sensitivity toward dinaciclib could be seen.

Summed up, we conclude that Cdk1/2/5/9 is heterogeneously expressed in BTC cells and dinaciclib exhibits strong anti-BTC properties in 2D and 3D cell culture. Additionally, we revealed that the reduction in tumor growth might be linked to the downregulation of proteins involved in tumor progression like EGFR and STAT3 and anti-apoptotic proteins like Mcl-1 via dinaciclib. Therefore, dinaciclib might be a promising new therapeutic option for the treatment of BTC patients.

## Methods

### Cell lines, substances

In the present study, a panel of ten established BTC cell lines was used: CCC-5 (ACC-810), EGI-1 (ACC-385) and TFK-1 (ACC-344) cells were purchased from the German Collection of Microorganisms and Cell Cultures (DSZM; Braunschweig, Germany); HuCCt-1 (JCRB0425), KKU-055 (JCRB1551), KKU-100 (JCRB1568), KKU-213 (JCRB1557), NOZ (JCRB1033), OCUG-1 (JCRB0191) and OZ (JCRB1032) were purchased from the Japanese Collection of Research Bioresources Cell Bank (JCRB; Osaka, Japan). The human cholangiocyte cell-line MMNK-1 (JCRB1554), purchased from the Japanese Collection of Research Bioresources Cell Bank, was used to evaluate the effects of dinaciclib in cholangiocytes. Cells were cultured in high-glucose Dulbecco’s Modified Eagle Medium (DMEM; ThermoFisher Scientific, Waltham, MA, USA) supplemented with 10% (v/v) fetal bovine serum (FBS; Biochrom, Berlin, Germany), 1% (v/v) antibiotic-antimycotic (ABAM; Merck, Darmstadt, Germany), 1 mm sodium pyruvate (Pan Biotech, Aidenbach, Germany) and 10 mm HEPES (Pan Biotech). Cells were cultured under standardized conditions (37°C in a humidified atmosphere containing 5% CO_2_). Cyclin-dependent kinase (Cdk) inhibitor dinaciclib as well as cisplatin were purchased from Selleckchem (Houston, TX, USA). Dinaciclib was dissolved in 100% dimethyl sulfoxide (DMSO), whereas cisplatin was dissolved in water.

### Viability/Cytotoxicity

Cells were seeded at 1 × 10^4^ cells in 100 µl growth medium in 96-well microplates, were grown for 24 h and washed with serum-free medium (sfDMEM). Cells were treated with dinaciclib in an 11-step dilution series (0.1–100 nM) in sfDMEM to avoid interactions between serum proteins and dinaciclib. Treated cells were incubated under standard culture conditions (5% CO_2_, 37°C) for 72 h. After incubation, cell viability was measured on a Spark multimode reader (Tecan, Grödig, Austria) after incubation with 0.5 mm resazurin for 1 h at 37°C (Alfa Aesar, Haverhill, MA, USA). Time-resolved viability analysis was investigated in KKU-100, OCUG-1 and OZ using the same seeding number and concentration conditions as above. Cell viability was measured after 0, 6, 24, 48 and 72 h. Viability was expressed as a percentage of the respective initial (0 h) value for each concentration.

### Immunoblot

Cells were seeded in 60 mm dishes with a final cell number of 1 × 10^6^ cells and allowed to grow overnight. After 24 h, cells were harvested, centrifuged, counted and diluted with PBS to a final cell concentration of 1 × 10^7^ cells per ml. Cells were lysed via sonification (Sonopuls HD70, UW70 ultrasound head, Bandelin) with 10–15 pulses and centrifuged for 5 min at 4°C. Supernatants were mixed 1:1 with 2X novex^TM^ tris-glycine sodium dodecyl sulfate sample buffer (Thermo Fisher Scientific; Vienna, Austria) and incubated at 95°C for 5 min. About 7.5 × 10^4^ cells per slot were loaded onto stain-free gradient gels (4–20% Mini-PROTEAN® TGX™ Gel (Bio-Rad, Hercules, US)) and run at 100 V for 60–80 min. Nitrocellulose membrane blots were incubated with primary antibodies: anti-Cdk1 (#77055, monoclonal, rabbit, 1:1000), anti-Cdk2 (#18048, monoclonal, rabbit, 1:1000), anti-Cdk5 (#2506, monoclonal, rabbit, 1:1000), anti-Cdk9 (#2316, monoclonal, rabbit, 1:1000), anti-AKT (#9272, polyclonal, rabbit, 1:1000), anti-EGFR (#4267, monoclonal, rabbit, 1:1000), anti-PTK2 (#71433, monoclonal, rabbit, 1:1000), anti-STAT3 (#9139, monoclonal, mouse, 1:1000), anti-ERK (#9102, polyclonal, rabbit, 1:1000), anti-Mcl-1 (#5453T, monoclonal, rabbit, 1:1000), anti-phospho-Mcl-1 (Ser64) (13297S, monoclonal, rabbit, 1:1000), anti-phospho-EGFR (Tyr1068) (#2234, monoclonal, rabbit, 1:1000), anti-phospho-STAT3 (Tyr705) (#9145S, monoclonal, rabbit, 1:1000), anti-phospho-ERK (Thr202/Tyr204) (#9106S, monoclonal, mouse, 1:1000); all purchased from Cell Signaling Technology, Frankfurt, Germany) overnight at 4°C. Blots were rinsed and incubated with a HRP-conjugated substrate (1:10000; Bio-Rad, Hercules, US) and secondary antibody: goat anti-rabbit IgG (H+L)-HRP conjugate (#1706515, 1:1000) or goat anti-mouse IgG (H+L)-HRP conjugate (#1706515, 1:1000), purchased from Bio-Rad (Hercules, US) for 1 h at room temperature. Chemiluminescent images were evaluated with the corresponding stain-free images. For baseline expression analysis (Cdk1, Cdk2, Cdk5 and Cdk9), a virtual reference value was used in order to make numerical results more comparable across biological replicates. This reference value was created by summing the band intensities for each biological replicate. Subsequently, the individual band intensity for each cell line was normalized to the summed intensity bands.

### ATP assay (cell titer Glo®)

Selected cell lines were seeded and treated as described above. After 72 h incubation, CellTiter-Glo® Reagent (Promega, Fitchburg, Madison, US) was added to the cells according to the manufacturer’s protocol. Luminescence was recorded with the Spark reader with an integration time of 1000 ms. Luminescence values were normalized to the mean luminescence signal of controls (ATP level in % control).

### Continuous impedance assay (xCelligence)

The effect of dinaciclib on BTC cell lines was continuously assessed using the xCELLigence system from Roche Diagnostics (Mannheim, Germany) for 72 h. KKU-100, OCUG-1, and OZ cells were seeded at 1 × 10^4^ cells per well in 100 µl growth medium in equilibrated 96-well E-plates. After 24 h, the cells were either treated with 3.125 nM, the cell-line specific IC_50_ concentration (KKU-100: 8 nM, OCUG-1: 33 nM, OZ: 7 nM), or 100 nM dinaciclib versus medium only (control). The impedance, which is a dimensionless parameter proportional to the cell number, was measured every hour and normalized to the values at the start of treatment.

### 3D cell culture (spheroids)

Spheroids were prepared with the forced floating method in 96-well U-bottom low attachment plates (Nunclon Sphera, Thermo Scientific) using standard growth medium. In each well, 500–7,000 cells/well were seeded, and the plates were centrifuged for 10 min at 350 g and further cultivated for 72 h at 37°C, 5% CO_2_ in a humidified atmosphere. The spheroids were exposed to dinaciclib in sfDMEM (6.25 to 100 nM) for 96 h, and spheroid formation/size was monitored and imaged before and after treatment using an CKX53SF microscope (Olympus, Vienna, Austria). Cell viability after 96 h exposure to dinaciclib was determined with the Promega CellTiter-Glo® 3D cell viability assay according to the manufacturer’s instructions.

### Quantitative real-time PCR analysis (RT-PCR)

BTC cells were seeded into 6-well plates at a density of 4 × 10^5^. After 24 h, cells were treated with the indicated concentration of dinaciclib (KKU-100: 4, 8, 100 nM, OCUG-1: 16.5, 33, 100 nM, OZ: 3.5, 7, 100 nM) for another 24 h. mRNA isolation of cell culture samples was performed using the Qiagen RNeasy Mini Kit (Qiagen, Hilden, Germany) according to the manufacturer’s protocol. The High-Capacity cDNA Reverse Transcription Kit (Applied Biosystems, Foster City, CA) was used to transcribe mRNA as described by the manufacturer. RT-PCR was executed with the CFX96 Touch Real-Time PCR Detection System (Bio-Rad Laboratories GmbH, Feldkirchen, Germany) using the SsoAdvanced Universal SYBR Green Supermix (Bio-Rad) and respective primers (primers and corresponding sequences are listed in Table S3). GAPDH was used as a housekeeping gene. To evaluate changes in mRNA levels, the ΔΔCT method was used. Changes in gene expression (fold changes) were shown in a biologically meaningful way as fold regulation, where fold changes greater than two indicate upregulation (fold changes equal fold regulation) and fold changes less than 0.5 indicate downregulation (fold regulation calculated as –(1/fold change)). Respective primer sequences are shown in Table S3.

### Immunohistochemistry of Cdk5, EGFR and STAT3 in BTC cell blocks

Cells were seeded in 60 mm dishes and let grown overnight. Cell blocks were prepared using a 1:1 mix of citrate plasma and Thromborel S (Siemens Healthcare, Marburg, Germany).

E-cadherin and vimentin as the most important epithelial and mesenchymal marker proteins, respectively, were measured by semi-quantitative immunohistochemistry (IHC) on all FFPE tissue specimens. In brief, 4 µm sections were mounted on glass slides, deparaffinized using graded alcohols, subjected to antigen retrieval at pH9 and stained using primary antibodies (mouse-monoclonal anti-E-cadherin and anti-vimentin, each ready-to use; Ventana Medical Systems, Basel, Switzerland) on a Benchmark Ultra (Ventana). Immunohistochemistry for EGFR and STAT3 was performed on the cell blocks with the three BTC cell lines KKU-100, OZ and OCUG-1. In brief, 4 µm sections were mounted on glass slides, deparaffinized with graded alcohol, specifically pretreated and stained with the primary antibodies (listed in Table S2) on a Benchmark Ultra platform (Ventana) with the ultraView Universal DAB Detection Kit (Ventana). Immunohistochemistry was assessed by assessing the extent (% positive cells) and intensity (0–3) of IHC staining on three different representative microscope fields and expressed semi-quantitatively using the QuickScore method by multiplying the extent and intensity (giving values between 0 and 300) for each field.^25^

### Flow cytometry

BTC cells were seeded into 6-well plates at a density of 4 × 10^5^. After 24 h, cells were treated with the cell-line specific IC_50_ concentration (KKU-100: 8 nM, OCUG-1: 33 nM, OZ: 7 nM), the highest lethal dinaciclib concentration (KKU-100: 50 nM, OCUG-1 and OZ: 100 nM) or left untreated (control) for 24 h. On the day of analysis, supernatant was collected, cells were washed with PBS and harvested. 1 × 10^6^ cells were further centrifuged, washed with PBS containing 1% Accutase (Pan Biotech) and again centrifuged before they were stained with Annexin V and 7-AAD. In brief, annexin binding buffer (BD Biosciences, New Jersey, US), Annexin V (Thermo Fisher Scientific) and 7-AAD (eBioscience, San Diego, California, US) were added to the cells and incubated for 12 min at RT. Flow cytometry was performed with the Beckman Coulter Gallios device (Brea, California, US). Data were analyzed with the Kaluza Software from Beckman Coulter.

### Statistics and data representation

All experiments were performed at least three times and were statistically evaluated using GraphPad Prism 9.4.0. Statistically relevant differences between the untreated control and the treated samples were determined with the aid of one-way analysis of variance (ANOVA). Pearson correlation analysis was done using Prism 9.4.0 (GraphPad Software, Boston, MA, USA). IC_50_ values for dinaciclib were calculated using four-parameter logistic regression with OriginPro 2023 (OriginLab, Northampton, MA, USA).

## Supplementary Material

Supplementary information_revised clean.docx

## Data Availability

The datasets generated during and/or analyzed during the current study are available from the corresponding author on reasonable request.
