# Supplementary material for: The Cdk inhibitor dinaciclib as a promising anti-tumorigenic agent in biliary tract cancer
Source: Cancer Biol Ther. 2024 Dec 12;25(1):2439057. doi: 10.1080/15384047.2024.2439057 (PMC11789727; doi:10.1080/15384047.2024.2439057)
Supplement: Supplementary information_revised clean.docx [file KCBT_A_2439057_SM5663.docx]

1 Supplementary information

**120**

**120**

**120**

**100**

**KKU-100**

**Cell viability [%UTC]**

**100**

**100**

**OZ**

**Cell viability [%UTC]**

**80 80 80**

**60 60 60**

**40 40 40**

**20 20 20**

**0 0 0**

**OCUG-1**

**Cell viability [%UTC]**

**UTC**

**0.1**

**0.2**

**0.4**

**0.8**

**1.56**

**3.125**

**6.25**

**12.5**

**25**

**50**

**100**

**UTC**

**0.1**

**0.2**

**0.4**

**0.8**

**1.56**

**3.125**

**6.25**

**12.5**

**25**

**50**

**100**

**UTC**

**0.1**

**0.2**

**0.4**

**0.8**

**1.56**

**3.125**

**6.25**

**12.5**

**25**

**50**

**100**

**Dinaciclib [nM] (- serum)**

**Dinaciclib [nM] (- serum)**

**Dinaciclib [nM] (- serum)**

**120**

**120**

**120**

**100**

**KKU-100**

**Cell viability [%UTC]**

**100**

**100**

**OZ**

**Cell viability [%UTC]**

**80 80 80**

**60 60 60**

**40 40 40**

**20 20 20**

**0 0 0**

**OCUG-1**

**Cell viability [%UTC]**

**UTC**

**0.1**

**0.2**

**0.4**

**0.8**

**1.56**

**3.125**

**6.25**

**12.5**

**25**

**50**

**100**

**UTC**

**0.1**

**0.2**

**0.4**

**0.8**

**1.56**

**3.125**

**6.25**

**12.5**

**25**

**50**

**100**

**UTC**

**0.1**

**0.2**

**0.4**

**0.8**

**1.56**

**3.125**

**6.25**

**12.5**

**25**

**50**

**100**

**Dinaciclib [nM] (+ serum)**

# 2

**Dinaciclib [nM] (+ serum)**

**Dinaciclib [nM] (+ serum)**

## Figure S1. Comparison of dinaciclib treatment in serum-free and serum containing media.

1. Cell viability of KKU-100, OCUG-1 and OZ cells after treatment with increasing dinaciclib concentrations
2. in medium with (+ serum) or without (- serum) serum for 72 h.

(A)

**CCC-5**

**KKU-100**


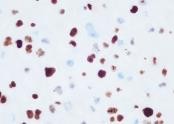

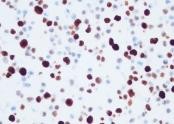

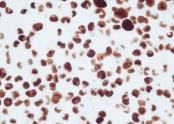

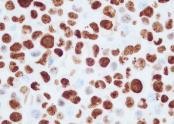

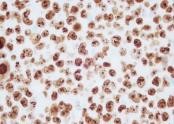

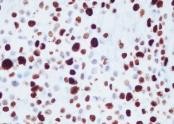

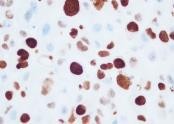

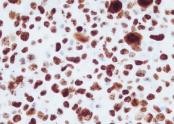


**50 µm**

**OCUG-1**

**EGI-1 HuCCT-1**

**KKU-213 MMNK-1**

**OZ TFK-1**

**KKU-055**

**NOZ**

**CCC-5 EGI-1 HuCCT-1 KKU-055 KKU-100 KKU-213 MMNK-1**

**NOZ OCUG-1**

**OZ TFK-1**

**0 20 40 60 80 100 120**


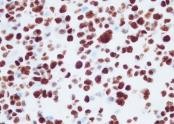

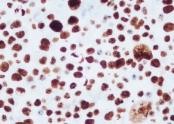

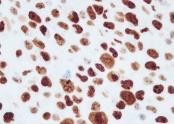


**IHC score**

(B)

**CCC-5**


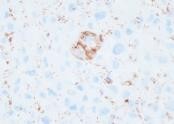

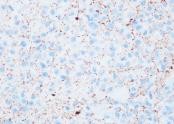

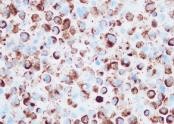

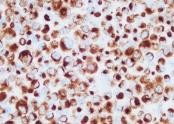


**50 µm**

**KKU-100**


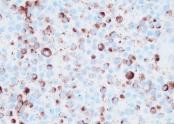

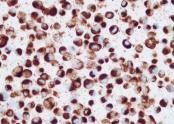

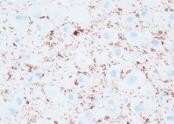

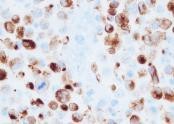


**OCUG-1**

**EGI-1 HuCCT-1**

**KKU-213 MMNK-1**

**OZ TFK-1**

**KKU-055**

**NOZ**

**CCC-5 EGI-1 HuCCT-1 KKU-055 KKU-100 KKU-213 MMNK-1**

**NOZ OCUG-1**

**OZ TFK-1**

**0 100 200 300 400**


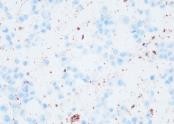

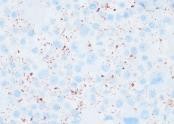

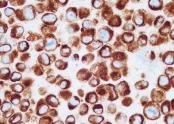


**IHC score**

(C)

**CCC-5**


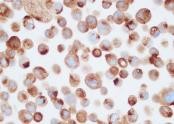

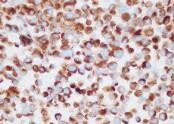

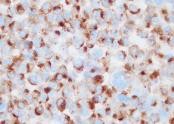

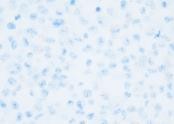


**50 µm**

**KKU-100**


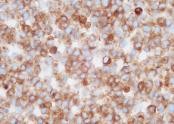

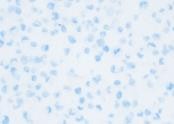

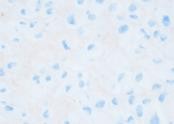

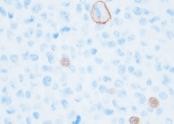


**OCUG-1**

**EGI-1 HuCCT-1**

**KKU-213 MMNK-1**

**OZ TFK-1**

**KKU-055**

**NOZ**

**CCC-5 EGI-1 HuCCT-1 KKU-055 KKU-100 KKU-213 MMNK-1**

**NOZ OCUG-1**

**OZ TFK-1**

**0 100 200 300**


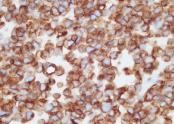

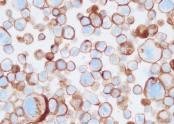

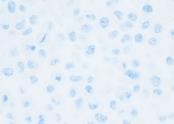


**IHC score**

# 6

## Figure S2. Various tumour markers are differently expressed in biliary tract cancer (BTC) cell lines.

1. Representative immunohistochemistry (IHC) pictures of (A) Ki67, (B) Vimentin and (C) E-cadherin
2. expression in different BTC cell lines. The bar charts show the IHC scores of the corresponding cell lines
3. and expressions.

### (A)


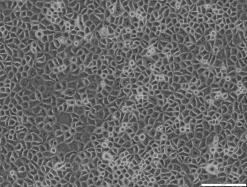

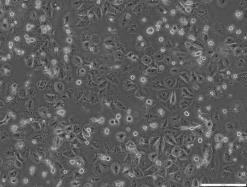

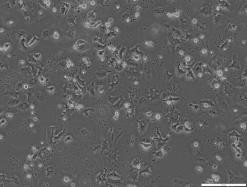

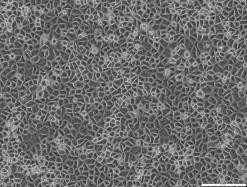

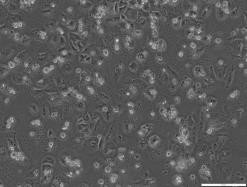

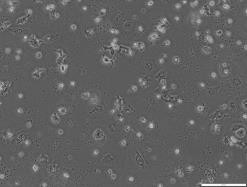

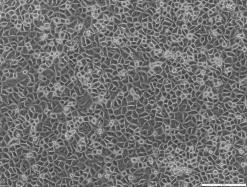

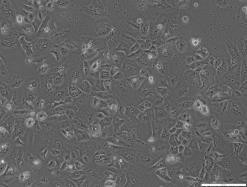

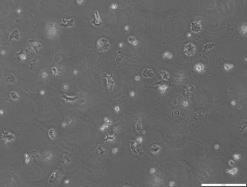

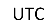

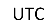

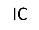

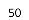

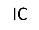

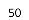

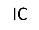

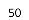

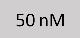

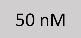

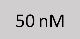

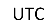


**24 h**

**KKU-100**

**48 h**

**72 h**

### (B)

**24 h**


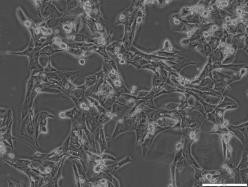

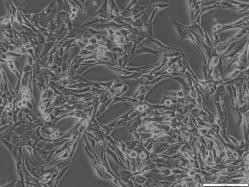

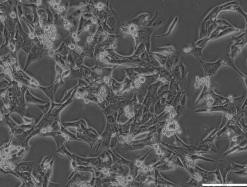

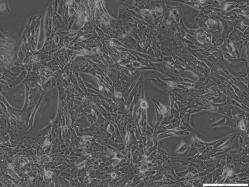

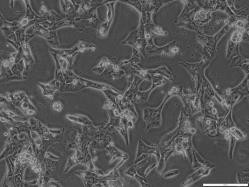

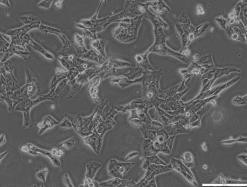

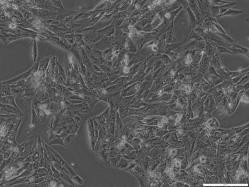

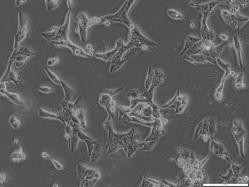

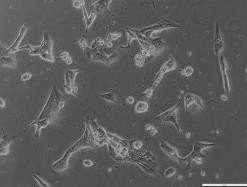

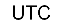

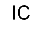

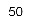

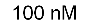

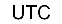

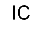

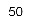

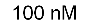

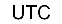

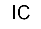

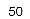

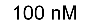


**OCUG-1**

**48 h**

**72 h**

### (C)

**24 h**


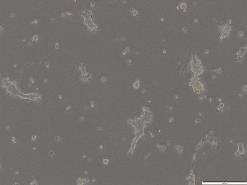

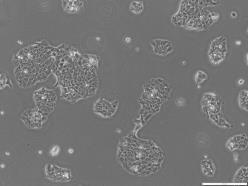

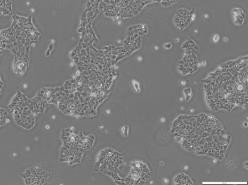

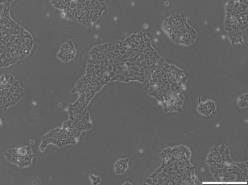

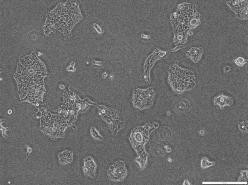

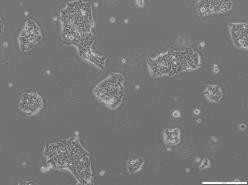

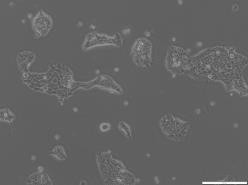

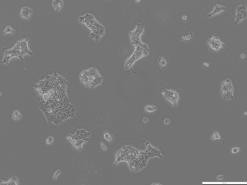

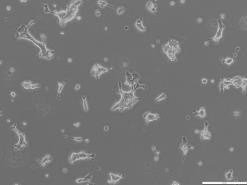


100 nM

IC50

UTC

100 nM

IC50

UTC

100 nM

IC50

UTC

**OZ**

**48 h**

**72 h**

# 11

## Figure S3. Dinaciclib causes morphological changes in cell shape.

1. Representative pictures of (A) KKU-100, (B) OCUG-1 and (C) OZ cells following dinaciclib treatment with
2. the respective IC50 (KKU-100: 8 nM, OCUG-1: 33 nM, OZ: 7 nM) and 50 / 100 nM dinaciclib (highest
3. concentration). Pictures were taken after 24, 48 and 72 h treatment with the Olympus CKX53SF
4. microscope, SC50 camera using a magnification of 20X.

# 17

### (A)

**125**

*

*

*

*

✱ ✱

✱ ✱

*

*

*

*

*

*

✱ ✱

✱ ✱

✱ ✱

✱ ✱

**100**


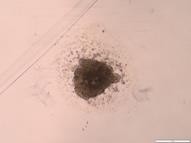

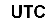

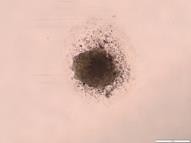

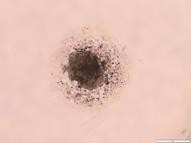

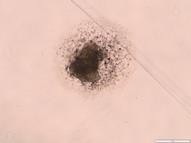

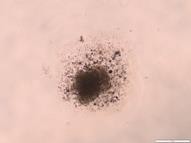

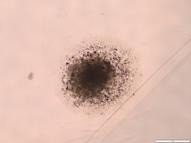

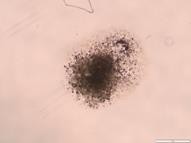

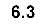

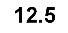

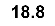

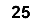

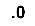

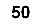

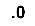

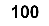


**CCC-5**

**Cell viability [% UTC]**

**75**

**50**

**25**

**0**

**UTC**

**6.3**

**12.5**

**18.8**

**25.0**

**50.0**

**100.0**

### (B)

**125**

**Dinaciclib [nM]**

**100**

*

*

✱ ✱

*

*

*

*

*

*

*

**EGI-1**

**Cell viability [% UTC]**

**75**

**50**

**25**

**0**

**UTC**

**9.4**

**12.5**

**18.8**

**25.0**

**50.0**

**Dinaciclib [nM]**

### (C)

**125**

**100**

**HuCCt-1**

**Cell viability [% UTC]**

**75**

**50**

**25**

**0**

**UTC**

**9.4**

**12.5**

**18.8**

**25.0**

**50.0**

### (D)

**Dinaciclib [nM]**

**150**

*

*

✱ ✱

✱ ✱

✱ ✱

*

*

✱ ✱

✱ ✱

✱ ✱

*

*

*

*

*

**125**

**100**

**KKU-213**

**Cell viability [% UTC]**

**75**

**50**

**25**

**0**

**UTC**

**6.3**

**9.4**

**12.5**

**18.8**

**25.0**

**50.0**

**100.0**

**Dinaciclib [nM]**

# 18

## Figure S4. Dinaciclib decreases the proliferation and size of 3D biliary tract cancer (BTC) spheroids.

1. Viable (A) CCC-5, (B) EGi-1, (C) HuCCt-1 and (D) KKU-213 cells after 96 h stimulation with the stated
2. dinaciclib concentrations measured with the Promega CellTiter-Glo® 3D cell viability assay. Exemplary
3. pictures of each treatment condition of BTC cells are shown (scale bar 500 µm), whereby the values
4. represent dinaciclib concentrations in nM. Data are presented as mean values ± SEM, n = 4 and
5. significances were tested using a one-way analysis of variance (ANOVA), Dunnett’s multiple
6. comparison test compared to the untreated control (UTC), *P<0.05, **P<0.01, ***P<0.001,
7. ****P<0.0001.

# 27

### (A)

**125**

*

✱ ✱

✱ ✱

✱ ✱

*

✱ ✱

✱ ✱

✱ ✱

✱ ✱

*

*

*

*

**100**

**MMNK-1**

**Cell viability [% UTC]**

**75**

**50**

**25**

**0**

**UTC**

**6.3**

**12.5**

**18.8**

**25.0**

**50.0**

**100.0**

**Dinaciclib [nM]**

### (B)

**125**

*

*

*

✱ ✱ ✱ ✱ ✱

✱ ✱ ✱ ✱ ✱

✱ ✱ ✱ ✱ ✱

✱ ✱ ✱ ✱ ✱

**100**

**NOZ**

**Cell viability [% UTC]**

**75**

**50**

**25**

**0**

**UTC**

**6.3**

**12.5**

**18.8**

**25.0**

**50.0**

**100.0**

**Dinaciclib [nM]**

### (C)

**125**

*

*

*

*

*

*

*

*

✱ ✱

✱ ✱

*

*

*

*

*

*

**100**

**TFK-1**

**Cell viability [% UTC]**

**75**

**50**

**25**

**0**

**UTC**

**6.3**

**12.5**

**18.8**

**25.0**

**50.0**

**100.0**

**Dinaciclib [nM]**

(D)

# 28

**KKU-055**

## Figure S5. Dinaciclib decreases the proliferation and size of 3D biliary tract cancer (BTC) spheroids.

1. Viable (A) MMNK-1, (B) NOZ and (C) TFK-1 cells after 96 h stimulation with the stated dinaciclib
2. concentrations measured with the Promega CellTiter-Glo® 3D cell viability assay. Exemplary pictures
3. of each treatment condition of BTC cells are shown (scale bar 500 µm), whereby the values represent
4. dinaciclib concentrations in nM. (D) Representative images of KKU-055 spheroids after 96 h of
5. Dinaciclib treatment with the indicated concentrations in nM. Data are presented as mean values ±
6. SEM, n = 4 and significances were tested using a one-way analysis of variance (ANOVA), Dunnett’s
7. multiple comparison test compared to the untreated control (UTC), *P<0.05, **P<0.01, ***P<0.001,
8. ****P<0.0001.

# 38

UTC

**0.0**

IC50

24 h

50.0 nM UTC IC50

48 h

50.0 nM

UTC

IC50

72 h

50.0 nM

UTC IC50

24 h

100.0 nM

UTC IC50

48 h

100.0 nM

UTC IC50

72 h

100.0 nM

UTC

IC50

24 h

100.0 nM

UTC

IC50

48 h

100.0 nM UTC

72 h

IC50

100.0 nM

**PTK2**

**Proteinexpression**

(C)

**[x-fold to UTC]**

**2.0**

**1.5**

**1.0**

**0.5**

✱ ✱ ✱ ✱

✱✱

**Proteinexpression [x-fold to UTC]**

**1.2**

**0.8**

**0.4**

**0.0**

*

**Proteinexpression [x-fold to UTC]**

**1.5**

**1.0**

**0.5**

**0.0**

UTC

IC50

**2.5**

50 nM UTC IC50

8.0 nM (IC_50_)

50.0 nM

50 nM

UTC IC50

50 nM

UTC IC50

**1.6**

100 nM

33.0 nM (IC_50_)

100.0 nM

UTC IC50

100 nM

UTC IC50

100 nM

UTC IC50

**2.0**

100 nM

7.0 nM (IC_50_)

100.0 nM

UTC IC50

100 nM UTC IC50

100 nM

**ERK**

**Proteinexpression**

**[x-fold to UTC]**

**1.0**

**0.5**

**0.0**

24 h

48 h

✱✱

✱✱

**Proteinexpression [x-fold to UTC]**

**1.0**

**0.5**

**0.0**

72 h

24 h

48 h

✱✱

*

✱ ✱ ✱

**Proteinexpression [x-fold to UTC]**

**1.0**

**0.5**

**0.0**

72 h

24 h

48 h

72 h

UTC

(B)

IC50

**1.5**

50.0 nM UTC IC50

8.0 nM (IC_50_)

50.0 nM

50.0 nM

UTC IC50

50.0 nM

UTC

**1.5**

IC50

100.0 nM

33.0 nM (IC_50_)

100.0 nM

UTC IC50

100.0 nM

UTC IC50

100.0 nM

UTC IC50

**1.5**

100.0 nM

7.0 nM (IC_50_)

100.0 nM

UTC

IC50

100.0 nM UTC

IC50

100.0 nM

**AKT**

**Proteinexpression**

**[x-fold to UTC]**

**1.0**

**0.5**

**0.0**

24 h

48 h

**Proteinexpression [x-fold to UTC]**

**1.5**

**1.0**

*

**0.5**

**0.0**

72 h

24 h

48 h

✱✱

**Proteinexpression [x-fold to UTC]**

**1.5**

**1.0**

**0.5**

**0.0**

72 h

24 h

48 h

72 h

**KKU-100**

8.0 nM (IC_50_)

50.0 nM

**OCUG-1**

33.0 nM (IC_50_)

100.0 nM

**OZ**

7.0 nM (IC_50_)

100.0 nM

(A)

**1.5**

**2.0**

**2.0**

39

9

## Figure S6. Dinaciclib affects the expression of proteins involved in cell proliferation and tumor

1. **progression.**
2. Shown is the effect of dinaciclib on (A) AKT, (B) ERK and (C) PTK2 protein expression and the
3. corresponding loading controls of KKU-100, OCUG-1 and OZ cells. Biliary tract cancer (BTC) cells were
4. either treated with the corresponding IC50 (KKU-100: 8 nM, OCUG-1: 33 nM, OZ: 7 nM), 50 nM (KKU-
5. 100) or 100 nM (OCUG-1 and OZ) dinaciclib for 24, 48 and 72 h, respectively. Protein expression was
6. quantified via normalization to corresponding loading controls, which were further referred to
7. untreated control cells [x-fold to untreated control = UTC]. Data are presented as mean values ± SEM,
8. n = at least 3 and significances were tested for each dinaciclib concentration against controls using a
9. one-way analysis of variance (ANOVA), Dunnett *P < 0.05, **P < 0.01, ***P < 0.001 and ****P < 0.0001.

# 50

10

**p-Mcl-1**

**40 kDa**

**Loading Control**

(D)

**40 kDa**

**Loading Control**

**Figure S7. Respective loading controls for Western blotting.**

Representative immunoblots probed with antibodies against (A) EGFR, (B) STAT3, (C) Mcl-1, (D)

phospho-Mcl-1 and corresponding loading controls are shown for each cell line (KKU-100, OCUG-1 and

OZ).

**Mcl-1**

**STAT3**

**EGFR**

UTC

IC50

50.0 nM

UTC IC50

50.0 nM

UTC IC50

50.0 nM

UTC

IC50

24 h

50.0 nM UTC IC50

48 h

50.0 nM

UTC

IC50

72 h

50.0 nM

UTC

**86 kDa**

**Loading Control**

(C)

IC50

24 h

50 nM UTC IC50

48 h

50 nM

UTC

IC50

72 h

50 nM

UTC

(A)

**175 kDa**

**Loading Control**

(B)

IC50

24 h

24 h

50 nM UTC IC50

48 h

50 nM

UTC

IC50

72 h

72 h

50 nM

UTC

24 h

IC50

100.0 nM

UTC

IC50

100.0 nM

UTC

IC50

100.0 nM

UTC IC50

100.0 nM UTC

48 h

72 h

IC50

100.0 nM

UTC IC50

100 nM

24 h

UTC IC50

48 h

100 nM

UTC IC50

72 h

100 nM

UTC IC50

100 nM

24 h

24 h

UTC IC50

48 h

100 nM

UTC IC50

72 h

72 h

100 nM

UTC

IC50

100.0 nM

UTC IC50

100.0 nM UTC

IC50

100.0 nM

UTC

IC50

24 h

100.0 nM

UTC

IC50

48 h

100.0 nM

UTC

IC50

72 h

100.0 nM

UTC IC50

100 nM

24 h

UTC

IC50

48 h

100 nM UTC IC50

72 h

100 nM

UTC IC50

100 nM

24 h

24 h

UTC

IC50

48 h

100 nM UTC IC50

72 h

72 h

100 nM

**KKU-100**

48 h

**OCUG-1**

48 h

**OZ**

48 h

51

52

53

54

55

56

11

UTC

**42,44 kDa**

**Loading Control**

**Figure S8. Dinaciclib affects the phosphorylation status of proteins involved in cell proliferation and**

IC50

50.0 nM

UTC IC50

50.0 nM

UTC IC50

50.0 nM

UTC IC50

100.0 nM UTC

IC50

100.0 nM UTC

IC50

100.0 nM

UTC

IC50

24 h

100.0 nM

UTC IC50

48 h

100.0 nM UTC

72 h

IC50

100.0 nM

**p-ERK**

**Proteinexpression [x-fold to UTC]**

**8**

**7**

**6**

**5**

**4**

**3**

**2**

**1**

**0**

24 h

48 h

**Proteinexpression [x-fold to UTC]**

**18**

**16**

**14**

**12**

**10**

**8**

**6**

**4**

**2**

**0**

72 h

24 h

48 h

**Proteinexpression [x-fold to UTC]**

7.0 nM (IC50)

100.0 nM

24 h

48 h

72 h

24 h

48 h

72 h

**6**

**4**

**2**

**0**

72 h

✱✱

✱ ✱ ✱

✱✱

✱✱

✱ ✱ ✱

*

✱✱

✱ ✱ ✱

✱✱

✱✱

✱✱

UTC

**86 kDa**

**Loading Control**

IC50

50.0 nM

8.0 nM (IC50)

50.0 nM

✱✱

UTC IC50

50.0 nM

UTC IC50

50.0 nM

UTC IC50

33.0 nM (IC50)

100.0 nM

100.0 nM

UTC IC50

100.0 nM

UTC IC50

100.0 nM

UTC

**10**

**8**

IC50

100.0 nM

7.0 nM (IC50)

100.0 nM

UTC IC50

✱ ✱ ✱

100.0 nM UTC

IC50

✱ ✱ ✱ ✱

100.0 nM

**p-STAT3**

**Proteinexpression [x-fold to UTC]**

n.b.

n.b.

**1.0**

**0.8**

**0.6**

**0.4**

**0.2**

**0.0**

24 h

48 h

(C)

✱✱

**Proteinexpression [x-fold to UTC]**

n.b.

n.b.

n.b.

**0.8**

**0.6**

**0.4**

**0.2**

**0.0**

72 h

24 h

48 h

✱ ✱ ✱ ✱

✱ ✱ ✱ ✱

✱ ✱ ✱ ✱

**Proteinexpression [x-fold to UTC]**

72 h

UTC

**175 kDa**

**Loading Control**

IC50

**1.4**

**1.2**

50.0 nM UTC

8.0 nM (IC50)

50.0 nM

IC50

50.0 nM

UTC

IC50

50.0 nM

UTC

**1.2**

**1.0**

IC50

100.0 nM UTC

33.0 nM (IC50)

100.0 nM

IC50

100.0 nM UTC

IC50

100.0 nM

UTC

**1.8**

**1.6**

**1.4**

**1.2**

**1.0**

**0.8**

**0.6**

**0.4**

**0.2**

**0.0**

IC50

100.0 nM

7.0 nM (IC50)

100.0 nM

UTC

IC50

100.0 nM

UTC

IC50

100.0 nM

**p-EGFR**

**Proteinexpression [x-fold to UTC]**

(A)

**100**

**90**

**80**

**70**

**60**

**50**

**40**

**30**

**20**

**10**

**0**

8.0 nM (IC50)

50.0 nM

24 h

48 h

(B)

✱ ✱ ✱

**Proteinexpression [x-fold to UTC]**

33.0 nM (IC50)

100.0 nM

**5**

**4**

**3**

**2**

**1**

**0**

24 h

48 h

72 h

✱ ✱ ✱

*

**Proteinexpression [x-fold to UTC]**

**1.4**

**1.2**

**1.0**

**0.8**

**0.6**

**0.4**

**0.2**

**0.0**

72 h

**KKU-100**

**OCUG-1**

**OZ**

57

58

59

**tumor progression.**

12

1. Shown is the effect of dinaciclib on (A) phospho-EGFR, (B) phospho-STAT3 and (C) phospho-ERK
2. phosphorylation status and the corresponding loading controls of KKU-100, OCUG-1 and OZ cells.
3. Biliary tract cancer (BTC) cells were either treated with the corresponding IC50 (KKU-100: 8 nM, OCUG-
4. 1: 33 nM, OZ: 7 nM), 50 nM (KKU-100) or 100 nM (OCUG-1 and OZ) dinaciclib for 24, 48 and 72 h,
5. respectively. Protein expression was quantified via normalization to corresponding loading controls,
6. which were further referred to untreated control cells [x-fold to untreated control = UTC]. Data are
7. presented as mean values ± SEM, n = at least 3 and significances were tested for each dinaciclib
8. concentration against controls using a one-way analysis of variance (ANOVA), Dunnett *P < 0.05, **P
9. < 0.01, ***P < 0.001 and ****P < 0.0001, n.b. = no band detected.

# 69

**1**

**00**

**80**

alive

early apoptotic late apoptotic necrotic

**60**

**40**

**20**

**0**

**UTC**

**IC_50_**

**50.0 / 100.0 nM**

**KKU-100**

**Annexin / 7AAD**

**UTC 8.0 nM (IC**50**) 50.0 nM**

**1**

**00**

**80**

alive

early apoptotic late apoptotic necrotic

**60**

**40**

**20**

**0**

**OCUG-1**

**Annexin / 7AAD**

**UTC 33.0 nM (IC**50**) 100.0 nM**

**1**

**00**

**80**

alive

early apoptotic late apoptotic necrotic

**60**

**40**

**20**

**0**

**OZ**

**Annexin / 7AAD**

**UTC 7.0 nM (IC**50**) 100.0 nM**

# 70

## Figure S9. Dinaciclib induces apoptosis in biliary tract cancer (BTC) cells.

1. Flow cytometry analysis revealed the ability of dinaciclib to induce apoptosis in biliary tract cancer
2. (BTC) cells. BTC cells were either treated with the corresponding IC50 (KKU-100: 8 nM, OCUG-1: 33 nM,
3. OZ: 7 nM), 50 nM (KKU-100), 100 nM (OCUG-1 and OZ) dinaciclib or left untreated for 24 h before they
4. were stained with Annexin V and 7-AAD. Shown are representative plots for each cell line and condition
5. and the quantified cell populations (bar charts) for n = at least 2 biological replicates.

# 77

**120**

OCUG-1 MMNK-1 KKU-213 EGI-1 KKU-055

NOZ

HuCCt-1 TFK-1 OZ

KKU-100

CCC-5

**100**

**80**

**Viability [% UTC]**

**60**

**40**

**20**

**0**

**0.1 1 10 100**

**Cisplatin [µM]**

# 78

## Figure S10. Cisplatin decreases the cell viability dose-dependent in 10 biliary tract cancer (BTC) and

1. **1 non-tumorigenic cell line.**
2. Cell viability of 10 malignant BTC cell lines and one non-tumorigenic (MMNK-1) cell line after treatment
3. with increasing cisplatin concentrations for 72 h. Lines represent the overall fit of the four-parameter
4. logistic regression.
5. **Table S1. Significances for cell viability analyses.**

| **Dinaciclib [nM]** | **0.10** | **0.20** | **0.39** | **0.78** | **1.56** | **3.13** | **6.25** | **12.50** | **25.00** | **50.00** | **100.00** |
| --- | --- | --- | --- | --- | --- | --- | --- | --- | --- | --- | --- |
| **CCC-5** | ns | ns | ns | ns | ns | ns | ns | ns | **** | **** | **** |
| **EGI-1** | ns | ns | ns | ns | ns | ns | **** | **** | **** | **** | **** |
| **HuCCt-1** | ns | ns | ns | ns | ns | ns | ns | **** | **** | **** | **** |
| **KKU-055** | ns | ns | ns | ns | ns | ns | ns | **** | **** | **** | **** |
| **KKU-100** | ns | ns | ns | ns | ns | ns | ns | **** | **** | **** | **** |
| **KKU-213** | ns | ns | ns | ns | ns | ns | * | **** | **** | **** | **** |
| **MMNK-1** | ns | ns | ns | ns | ns | ns | **** | **** | **** | **** | **** |
| **NOZ** | ns | ns | ns | ns | ns | ns | ** | **** | **** | **** | **** |
| **OCUG-1** | ns | ns | ns | ns | ns | ns | * | ** | **** | **** | **** |
| **OZ** | ns | ns | ns | ns | ns | ns | ** | **** | **** | **** | **** |
| **TFK-1** | ns | ns | ns | ns | ns | ns | ns | **** | **** | **** | **** |

# 85

1. Significances were calculated with GraphPad Prism using one-way ANOVA. *P < 0.1, **P < 0.01,
2. ***P < 0.001, ****P < 0.0001 and ns (not significant). Significances corresponding to Figure 1A.

# 88

89 **Table S2. Antibodies used for immunohistochemistry and the respective additional information.**

90

| **Antibody** | **Species** | **Clone** | **Vendor** | **Dilution** | **Pretreatment**  **conditions** | **Incubation** |
| --- | --- | --- | --- | --- | --- | --- |
| EGFR | Mouse | 3C6 | Roche | Ready-to-  use (rtu) | Protease for 8 min | 32 min |
| STAT3 | Mouse | 124H6 | Cell  Signaling | 1:200 | High pH for 64 min at  95°C | 32 min |

91

| **Antibody** | **Vendor** | **Cat.-No.** | **Clone** | **Pre-**  **treatment** | **Dilution/**  **Incubation** | **Detection**  **kit** | **Platform** |
| --- | --- | --- | --- | --- | --- | --- | --- |
| Cdk5 | Abcam | ab40773 | EP715Y | High pH | 1:100 | OptiView | Ventana |
| E-Cadherin | Ventana | 05905290001 | 36 | High pH | rtu | UltraView | Ventana |
| Ki67 | Ventana | 05278384001 | 30-9 | High pH | rtu | UltraView | Ventana |
| Vimentin | Ventana | 05278139001 | V9 | High pH | rtu | UltraView | Ventana |

92

93 **Table S3. Primer sequences for RT-PCR.**

| **Gene** | **Sequence (5‘-3‘)** |
| --- | --- |
| AKT fwd | AAGTACTCTTTCCAGACCC |
| AKT rev | TTCTCCAGCTTGAGGTC |
| EGFR fwd | GGCCTAAGATCCCGTCCATC |
| EGFR rev | TGGCTTTCGGAGATGTTGCT |
| ERK fwd | GAAGCATTATCTTGACCAGC |
| ERK rev | TCCATGGCACCTTATTTTTG |
| GAPDH fwd | TGTTCGTCATGGGTGTGAACC |
| GAPDH rev | GCAGTGATGGCATGGACTGTG |
| PTK2 fwd | GTATGTCCCTATGGTGAAGG |
| PTK2 rev | GGTCAGAGTTCAATAGCTTC |
| STAT3 fwd | GGTACATCATGGGCTTTATC |
| STAT3 rev | TTTGCTGCTTTCACTGAATC |

94
